# Incidence of depression in patients with psoriasis and psoriatic arthritis treated with biologic therapy: Protocol for a systematic review and meta-analysis

**DOI:** 10.1371/journal.pone.0351646

**Published:** 2026-06-29

**Authors:** Emily Sirotich, Weston Lowry, Shi-Yi Wang, Madison Jurgens, Jeffrey M. Cohen

**Affiliations:** 1 Yale School of Medicine, New Haven, Connecticut, United States of America; 2 Yale Cancer Outcomes, Public Policy, and Effectiveness Research (COPPER) Center, Yale School of Medicine, New Haven, Connecticut, United States of America; 3 Department of Chronic Disease Epidemiology, Yale School of Public Health, New Haven, Connecticut, United States of America; 4 Harvey Cushing/John Hay Whitney Medical Library, Yale University, New Haven, Connecticut, United States of America; 5 Department of Dermatology, Yale School of Medicine, Yale University, New Haven, Connecticut, United States of America; 6 Department of Biomedical Informatics and Data Science, Yale School of Medicine, New Haven, Connecticut, United States of America; Bogomolets National Medical University: Nacional’nij medicnij universitet imeni O O Bohomol’ca, UKRAINE

## Abstract

**Background:**

Psoriasis and psoriatic arthritis are chronic immune-mediated diseases with both physical and psychological consequences. Depression is a common comorbidity associated with impaired quality of life and lower treatment adherence. Biologics, monoclonal antibodies targeting key cytokines, may favorably influence depressive symptoms by reducing systemic inflammation and improving disease activity. However, current evidence is heterogeneous and has not been comprehensively synthesized.

**Methods:**

This systematic review and meta-analysis will evaluate whether biologic therapy reduces the risk of depression among adults with psoriasis or psoriatic arthritis compared with non-biologic treatment or no therapy. Electronic searches will include MEDLINE, Embase, Cochrane Library, PsycINFO, and ClinicalTrials.gov. Eligible designs are randomized controlled trials (RCTs), cohort, and case–control studies reporting depression incidence or prevalence. Depression may be ascertained through clinical diagnosis or validated instruments. Study selection, data extraction, and risk-of-bias assessment will be performed independently by two reviewers. Using the Cochrane Risk of Bias 2.0 tool and the Newcastle–Ottawa Scale, we will assess quality of each study. Data permitting, random-effects meta-analysis will estimate pooled risk ratios (RRs) or odds ratios (ORs) with 95% confidence intervals (CIs). Certainty of evidence will be rated using the Grading of Recommendations, Assessment, Development and Evaluation (GRADE) approach.

**Discussion:**

This review will synthesize the impact of biologic therapy on incident depression in psoriasis and psoriatic arthritis, informing integrated care for patients with psoriatic disease.

**Systematic review registration:**

PROSPERO (submitted; registration number to be confirmed prior to data extraction).

## Introduction

Psoriasis and psoriatic arthritis are chronic immune-mediated diseases that substantially affect both physical and psychological health [1]. Depression is one of the most common comorbidities, contributing to reduced quality of life, social isolation, and poorer treatment adherence. A recent systematic review concluded that depression and suicidal ideation are significantly more prevalent among patients with psoriasis than the general population [1]. The study highlighted the psychosocial burden of psoriasis but did not include data on how treatment may impact this association.

Multiple studies have demonstrated that the systemic inflammation underlying psoriasis may share biological pathways with depression, including dysregulation of proinflammatory cytokines such as tumor necrosis factor (TNF), interleukin (IL)-17, and IL-23 [2,3]. Biologics are monoclonal antibodies that directly target inflammatory mediators implicated in both psoriasis and depression. In addition, reduction in depression could be associated with symptomatic improvement of psoriasis. However, current evidence from observational and clinical studies is heterogeneous and has not yet been synthesized comprehensively [4–6].

This systematic review with meta-analysis aims to evaluate whether biologic therapy lowers the incidence of depression among patients with psoriasis and psoriatic arthritis compared with non-biologic treatments.

The primary objective of this systematic review is to determine whether the use of biologic therapies is associated with a decreased incidence or risk of depression in adults with psoriasis or psoriatic arthritis compared with patients receiving non-biologic treatment or no therapy. Specifically, we aim to assess whether biologic agents targeting TNF, IL-12, IL-17, or IL-23 pathways reduce clinically diagnosed depression or depressive symptom scores.

### PICO framework

The study question is structured using the PICO framework as described in Table 1.

**Table 1:** PICO framework for the systematic review.

Component	Description
Population (P)	Adults ≥ 18 years with psoriasis or psoriatic arthritis
Intervention (I)	Biologic therapies (TNF, IL-12, IL-17, IL-23 inhibitors, etc.)
Comparator (C)	Non-biologic therapy or no treatment
Outcome (O)	Depression incidence or depressive symptom scores

## Materials and methods

This protocol has been submitted to the PROSPERO database (registration number to be confirmed) prior to initiation of data extraction to ensure transparency and to prevent duplication of research efforts. No results have been generated for this study and the protocol is submitted prior to data collection. This protocol follows the Preferred Reporting Items for Systematic Review and Meta-Analysis Protocols (PRISMA-P) reporting guidelines (S2 Appendix) [7,8]. The anticipated timeline is data collection and extraction completed by May 2026 and analysis completed by July 2026. Ethics approval is not applicable.

### Eligibility criteria

We will include studies involving adult participants aged 18 years or older diagnosed with psoriasis or psoriatic arthritis. Eligible study designs will include randomized controlled trials (RCTs), cohort studies, and case–control studies that compare biologic interventions with non-biologic treatments or no therapy and report depression as an outcome. We will include studies published in peer-reviewed journals. Studies must report data on at least 10 participants to be eligible for inclusion.

Depression may be ascertained through clinical diagnosis (e.g., International Classification of Diseases [ICD] or Diagnostic and Statistical Manual of Mental Disorders [DSM] codes, physician-documented diagnosis) or through validated self-report instruments (e.g., Patient Health Questionnaire-9 [PHQ-9], Beck Depression Inventory [BDI], Montgomery–Åsberg Depression Rating Scale [MADRS], Hamilton Depression Rating Scale [HAM-D]). Studies using either method of ascertainment will be eligible.

Studies will be excluded if they involve pediatric populations, are case reports, reviews, editorials, or conference abstracts lacking sufficient data, or if depression was not reported as an outcome. Additionally, studies including fewer than 10 individuals will be excluded.

### Information sources

The reporting of this systematic review protocol was guided by the standards of the PRISMA-P 2015 statement [8]. The following electronic databases will be searched: Ovid MEDLINE ALL, APA PsycInfo (Ovid), Embase (Ovid), Web of Science Core Collection, ClinicalTrials.gov, CENTRAL (Cochrane Library), and PubMed (NCBI).

An experienced medical librarian (MJ) will be consulted on methodology and a medical subject heading (MeSH) analysis of known articles provided by the research team will be done using the Yale MeSH Analyzer [9]. Scoping searches will be done in each database and an iterative process will be used to translate and refine the searches. To maximize sensitivity, the formal search will use controlled vocabulary terms and synonymous keywords to capture the concepts of “psoriasis/ psoriatic arthritis,” “biologic therapy,” and “depression.” No date or language limits will be imposed on the search. The search strategy will be peer reviewed by a second librarian, not otherwise associated with the project, using the PRESS standard [10]. A draft MEDLINE search strategy is provided in S1 Appendix.

Reviewers will check for additional relevant cited and citing articles using included studies. To capture recently published articles, a second database search will be rerun before publishing the paper. The reference lists of included articles and relevant reviews will also be screened manually to identify additional studies.

Search results will be pooled in EndNote 21 [11] and de-duplicated using the Reference Deduplicator tool [12]. This set will be uploaded to Covidence for screening [13]. Two screeners will independently review the titles, abstracts, and full text of the eligible articles that meet inclusion criteria. Any conflicts will be resolved through consensus.

### Search strategy

A draft MEDLINE search strategy is provided in S1 Appendix. The strategy will be adapted for each database.

### Study selection

All citations retrieved from the search will be imported into Covidence software for reference management and removal of duplicates. Two reviewers will independently screen titles and abstracts to identify potentially eligible studies. Full texts of selected articles will then be reviewed against inclusion and exclusion criteria. Disagreements will be resolved through discussion or adjudication by a third reviewer. The study selection process will be documented in a PRISMA flow diagram, including reasons for study exclusion at each stage.

### Data collection process

Data extraction will be performed independently by two reviewers using a standardized electronic form. Extracted information will include details of study design, author and publication year, country, sample size, demographics, intervention characteristics (including type, dose, and duration of biologic therapy), comparator, and outcomes related to depression. We will extract effect measures—such as risk ratio (RR), odds ratio (OR), or hazard ratio (HR) with corresponding 95% confidence intervals—and note any confounding factors adjusted for in the original studies. Information about study funding sources and declared conflicts of interest will also be recorded.

To address potential heterogeneity in depression ascertainment across studies, we will record the specific method used to identify depression in each study. Studies will be categorized according to whether depression was ascertained via clinical diagnosis (ICD/DSM codes or physician documentation) or via validated self-report instruments (e.g., PHQ-9, BDI, MADRS, HAM-D). Where possible, separate pooled estimates will be calculated for each category. Additionally, we will extract data on the threshold or cut-off score used for each instrument.

Key confounders that will be extracted for each study include baseline disease severity (e.g., Psoriasis Area and Severity Index [PASI] score), prior history of depression or other psychiatric comorbidity, concomitant use of psychotropic medications, and disease duration. We will note which confounders were adjusted for in the original analyses and will conduct sensitivity analyses restricted to studies that controlled for these variables.

Key data fields include: PICO elements; study design and quality indicators; type of biologic (TNF, IL-12, IL-17, IL-23 inhibitor, etc.); method of depression ascertainment; depression incidence; depression prevalence; follow-up duration; confounders controlled for; and funding source.

### Outcomes

The primary outcome of interest is the risk of depression following initiation of biologic versus non-biologic or no treatment. Secondary outcomes will include changes in mean depressive symptom scores over time and association between treatment response and mean difference in depressive symptom scores. Treatment response will be defined according to each study.

### Risk of bias assessment

Risk of bias for individual studies will be assessed using appropriate validated tools. For randomized controlled trials, we will employ the Cochrane Risk of Bias 2.0 instrument. For observational studies, we will use the ROBINS-I, which evaluates selection, comparability, and outcome or exposure assessment. Discrepancies between reviewers will be resolved by consensus.

### Data synthesis

Where sufficient data are available and outcomes are judged to be clinically and methodologically homogeneous, a quantitative synthesis will be conducted. Random-effects meta-analysis using the DerSimonian–Laird method will be performed to account for variation in effect sizes. Pooled measures will be expressed as risk ratios, odds ratios, or standardized mean differences with 95% confidence intervals. Heterogeneity will be quantified using the I² statistic.

If data are too heterogeneous to pool, findings will be synthesized narratively, emphasizing characteristics and direction of effects across studies. Planned subgroup analyses include: class of biologic (TNF, IL-12, IL-17, or IL-23 inhibitor); concomitant psoriatic arthritis; study design (RCT vs. observational); type of comparator (non-biologic therapy vs. no treatment, analyzed separately where data permit); and method of depression ascertainment (clinical diagnosis vs. validated instrument). Sensitivity analyses will be performed excluding studies judged to have high risk of bias and restricting to studies that adjusted for key confounders (baseline disease severity, prior psychiatric history, and psychotropic medication use). Also, if the outcome is dichotomous (yes or no depression), we will calculate odds ratio, with subgroup analysis by these two groups. If outcome is continuous, we will then calculate standardized mean difference across all instruments.

### Meta-bias assessment

Potential publication bias will be evaluated through funnel-plot visualization and Egger’s regression test when at least ten studies are available. Selective reporting bias will be assessed by comparing outcomes listed in registered protocols or trial registries with published results.

### Certainty of evidence

The overall certainty across outcomes will be assessed using the Grading of Recommendations, Assessment, Development and Evaluation (GRADE) approach [14]. The certainty of evidence will be categorized as high, moderate, low, or very low based on considerations of risk of bias, consistency, directness, precision, and publication bias.

## Discussion

This systematic review will synthesize and evaluate the available evidence to assess whether biologic therapy influences the incidence or severity of depression in patients with psoriasis and psoriatic arthritis. Integration of psychosocial outcomes into dermatologic and rheumatologic management remains a major unmet need, and this review aims to inform both clinicians and researchers. By examining the relationship between biologic treatment and mental health outcomes, the study will contribute to a more comprehensive understanding of the systemic and psychological dimensions of psoriatic disease.

Potential limitations include heterogeneity in depression definitions, variability in depression measurement tools, differences in study populations, and confounding by baseline disease severity. Additionally, real-world data may be influenced by indication bias if biologic therapy is preferentially prescribed to certain patient subgroups. Despite these challenges, this protocol adheres to PRISMA-P and GRADE guidelines and employs rigorous methodology to ensure transparency and reproducibility.

## Supporting information

S1 Appendix Draft MEDLINE search strategy. The complete search strategy developed for Ovid MEDLINE ALL, including controlled vocabulary terms and keywords for psoriasis, biologic therapy, and depression.
(DOCX)

S2 Appendix PRISMA- P Checklist. (DOCX)
