# Supplementary material for: Incidence of depression in patients with psoriasis and psoriatic arthritis treated with biologic therapy: Protocol for a systematic review and meta-analysis
Source: PLoS One. 2026 Jun 29;21(6):e0351646. doi: 10.1371/journal.pone.0351646 (PMC13313330; doi:10.1371/journal.pone.0351646)
Supplement: S1 Appendix — The complete search strategy developed for Ovid MEDLINE ALL, including controlled vocabulary terms and keywords for psoriasis, biologic therapy, and depression. (DOCX) [file pone.0351646.s001.docx]

August 12

Search History

(14searches found)

| \|  \| [# ▲](https://ovidsp.dc2.ovid.com/ovid-new-a/ovidweb.cgi?&S=3597d89e-ef44-4533-a788-5b7382740a57%7cmain&Sort+Sets=descending) \| Searches \| Results \| Runtime \| Type \| Actions \| Annotations \| \| --- \| --- \| --- \| --- \| --- \| --- \| --- \| --- \| \|  \| \| \| \| \| \| \|  \| \|  \| 1 \| Depression/ \| 172373 \| 0.08 \| Advanced \| [Display Results](https://ovidsp.dc2.ovid.com/ovid-new-a/ovidweb.cgi?&S=3597d89e-ef44-4533-a788-5b7382740a57%7cmain&SELECT=S.sh%7c&R=1&Process+Action=display)  More \|  \| \|  \| 2 \| Psoriasis/ \| 45756 \| 0.00 \| Advanced \| [Display Results](https://ovidsp.dc2.ovid.com/ovid-new-a/ovidweb.cgi?&S=3597d89e-ef44-4533-a788-5b7382740a57%7cmain&SELECT=S.sh%7c&R=2&Process+Action=display)  More \|  \| \|  \| 3 \| 1 and 2 \| 365 \| 0.07 \| Advanced \| [Display Results](https://ovidsp.dc2.ovid.com/ovid-new-a/ovidweb.cgi?&S=3597d89e-ef44-4533-a788-5b7382740a57%7cmain&SELECT=S.sh%7c&R=3&Process+Action=display)  More \|  \| \|  \| 4 \| exp Biological Products/ \| 742782 \| 1.96 \| Advanced \| [Display Results](https://ovidsp.dc2.ovid.com/ovid-new-a/ovidweb.cgi?&S=3597d89e-ef44-4533-a788-5b7382740a57%7cmain&SELECT=S.sh%7c&R=4&Process+Action=display)  More \|  \| \|  \| 5 \| 3 and 4 \| 12 \| 0.04 \| Advanced \| [Display Results](https://ovidsp.dc2.ovid.com/ovid-new-a/ovidweb.cgi?&S=3597d89e-ef44-4533-a788-5b7382740a57%7cmain&SELECT=S.sh%7c&R=5&Process+Action=display)  More \|  \| \|  \| 6 \| psoriasis.mp. [mp=title, book title, abstract, original title, name of substance word, subject heading word, floating sub-heading word, keyword heading word, organism supplementary concept word, protocol supplementary concept word, rare disease supplementary concept word, unique identifier, synonyms, population supplementary concept word, anatomy supplementary concept word] \| 65474 \| 0.89 \| Advanced \| [Display Results](https://ovidsp.dc2.ovid.com/ovid-new-a/ovidweb.cgi?&S=3597d89e-ef44-4533-a788-5b7382740a57%7cmain&SELECT=S.sh%7c&R=6&Process+Action=display)  More \|  \| \|  \| 7 \| psoriatic.mp. [mp=title, book title, abstract, original title, name of substance word, subject heading word, floating sub-heading word, keyword heading word, organism supplementary concept word, protocol supplementary concept word, rare disease supplementary concept word, unique identifier, synonyms, population supplementary concept word, anatomy supplementary concept word] \| 26024 \| 0.19 \| Advanced \| [Display Results](https://ovidsp.dc2.ovid.com/ovid-new-a/ovidweb.cgi?&S=3597d89e-ef44-4533-a788-5b7382740a57%7cmain&SELECT=S.sh%7c&R=7&Process+Action=display)  More \|  \| \|  \| 8 \| 2 or 6 or 7 \| 74159 \| 0.05 \| Advanced \| [Display Results](https://ovidsp.dc2.ovid.com/ovid-new-a/ovidweb.cgi?&S=3597d89e-ef44-4533-a788-5b7382740a57%7cmain&SELECT=S.sh%7c&R=8&Process+Action=display)  More \|  \| \|  \| 9 \| depression.mp. [mp=title, book title, abstract, original title, name of substance word, subject heading word, floating sub-heading word, keyword heading word, organism supplementary concept word, protocol supplementary concept word, rare disease supplementary concept word, unique identifier, synonyms, population supplementary concept word, anatomy supplementary concept word] \| 560489 \| 0.48 \| Advanced \| [Display Results](https://ovidsp.dc2.ovid.com/ovid-new-a/ovidweb.cgi?&S=3597d89e-ef44-4533-a788-5b7382740a57%7cmain&SELECT=S.sh%7c&R=9&Process+Action=display)  More \|  \| \|  \| 10 \| 1 or 9 \| 560489 \| 0.09 \| Advanced \| [Display Results](https://ovidsp.dc2.ovid.com/ovid-new-a/ovidweb.cgi?&S=3597d89e-ef44-4533-a788-5b7382740a57%7cmain&SELECT=S.sh%7c&R=10&Process+Action=display)  More \|  \| \|  \| 11 \| 8 and 10 \| 1477 \| 0.04 \| Advanced \| [Display Results](https://ovidsp.dc2.ovid.com/ovid-new-a/ovidweb.cgi?&S=3597d89e-ef44-4533-a788-5b7382740a57%7cmain&SELECT=S.sh%7c&R=11&Process+Action=display)  More \|  \| \|  \| 12 \| biologic.mp. [mp=title, book title, abstract, original title, name of substance word, subject heading word, floating sub-heading word, keyword heading word, organism supplementary concept word, protocol supplementary concept word, rare disease supplementary concept word, unique identifier, synonyms, population supplementary concept word, anatomy supplementary concept word] \| 78360 \| 0.49 \| Advanced \| [Display Results](https://ovidsp.dc2.ovid.com/ovid-new-a/ovidweb.cgi?&S=3597d89e-ef44-4533-a788-5b7382740a57%7cmain&SELECT=S.sh%7c&R=12&Process+Action=display)  More \|  \| \|  \| 13 \| 4 or 12 \| 813073 \| 0.10 \| Advanced \| [Display Results](https://ovidsp.dc2.ovid.com/ovid-new-a/ovidweb.cgi?&S=3597d89e-ef44-4533-a788-5b7382740a57%7cmain&SELECT=S.sh%7c&R=13&Process+Action=display)  More \|  \| \|  \| 14 \| 10 and 11 and 13 \| 131 \| 0.06 \| Advanced \| [Display Results](https://ovidsp.dc2.ovid.com/ovid-new-a/ovidweb.cgi?&S=3597d89e-ef44-4533-a788-5b7382740a57%7cmain&SELECT=S.sh%7c&R=14&Process+Action=display)  More \|  \| |
| --- | --- | --- | --- | --- | --- | --- | --- | --- | --- | --- | --- | --- | --- | --- | --- | --- | --- | --- | --- | --- | --- | --- | --- | --- | --- | --- | --- | --- | --- | --- | --- | --- | --- | --- | --- | --- | --- | --- | --- | --- | --- | --- | --- | --- | --- | --- | --- | --- | --- | --- | --- | --- | --- | --- | --- | --- | --- | --- | --- | --- | --- | --- | --- | --- | --- | --- | --- | --- | --- | --- | --- | --- | --- | --- | --- | --- | --- | --- | --- | --- | --- | --- | --- | --- | --- | --- | --- | --- | --- | --- | --- | --- | --- | --- | --- | --- | --- | --- | --- | --- | --- | --- | --- | --- | --- | --- | --- | --- | --- | --- | --- | --- | --- | --- | --- | --- | --- | --- | --- | --- | --- | --- | --- | --- | --- | --- | --- | --- |

Combine with:

[Contract](https://ovidsp.dc2.ovid.com/ovid-new-a/ovidweb.cgi?&S=3597d89e-ef44-4533-a788-5b7382740a57%7cmain&SELECT=S.sh%7c&Contract=1&Main+Search+Page=Main+Search+Page)
